# Supplementary material for: Factors Related to Mpox-Vaccine Uptake among Men Who Have Sex with Men in Taiwan: Roles of Information Sources and Emotional Problems
Source: Vaccines (Basel). 2024 Mar 20;12(3):332. doi: 10.3390/vaccines12030332 (PMC10975344; doi:10.3390/vaccines12030332)
Supplement: Supplementary file 1 [file vaccines-12-00332-s001.zip › vaccines-2851954-supplementary.pdf]

Supplementary Table S1. Factors related to mpox vaccine uptake: Multivariable logistic regression models (N = 389).

	Receiving a Mpox vaccination	
	OR (95% CI)	*p*
5-year increment of age	1.430 (1.186–1.725)	<0.001
Sexual orientation [a]	0.923 (0.459–1.85)	0.822
Education level [b]	0.851 (0.375–1.929)	0.699
Anxiety	1.019 (0.976–1.064)	0.398
Depression	0.931 (0.878–0.986)	0.015
Perceived risk of contracting mpox	1.343 (1.009–1.786)	0.043
Receiving mpox information from social media	0.693 (0.337–1.424)	0.318
Receiving mpox information from healthcare providers	5.339 (3.322–8.581)	<0.001

CI: confidence interval; OR: odds ratio, [a]gay as reference; [b]senior high school or below as reference.
